# The relationship of sociocultural beliefs and infertile couples' attitude toward reproductive donation: A descriptive-correlational study

**DOI:** 10.18502/ijrm.v17i5.4599

**Published:** 2019-06-26

**Authors:** Robab Latifnejad Roudsari, Hamideh Jafari, Ali Taghipour

**Affiliations:** ^1^ Nursing and Midwifery Care Research Centre, Mashhad University of Medical Sciences, Mashhad, Iran.; ^2^ Student Research Committee, School of Nursing and Midwifery, Mashhad University of Medical Sciences, Mashhad, Iran.; ^3^ Social Determinants of Health, Research Centre, Mashhad University of Medical Sciences, Mashhad, Iran.

**Keywords:** Sociocultural beliefs, Attitude, Assisted reproductive techniques.

## Abstract

**Background:**

There are controversial views on accepting a reproductive donation in the world.

**Objective:**

This study aimed to determine the relationship between the sociocultural beliefs and infertile couples' attitude toward reproductive donation in Mashhad, Iran.

**Materials and Methods:**

This descriptive correlational study was conducted out on 115 infertile couples visiting Milad Infertility Center in Mashhad, Iran with using convenience sampling. The research instruments were valid and reliable sociocultural beliefs and attitude questionnaires, which were completed by the respondents on a self-report basis.

**Results:**

The mean score of attitude toward reproductive donation in infertile women and men was 58.3 ± 12.6 and 57.8 ± 12.0, and the mean score of sociocultural beliefs in infertile women and men was 67.7 ± 11.5 and 67.6 ± 12.4, respectively. There was a direct correlation between sociocultural beliefs and attitude toward reproductive donation in infertile women (p < 0.001) and men (p < 0.001), that is, women and men with a higher score of sociocultural beliefs had a higher score of attitude as well. A direct correlation was also seen between sociocultural beliefs and infertile women and men's public attitude, their attitude towards genetic bound between parents and children, their attitude regarding the issue of confidentiality of the donation process, as well as attitude in relation to oocyte donation, embryo donation, and surrogacy (p < 0.001).

**Conclusion:**

The findings suggest that sociocultural beliefs that surround reproductive donation could influence infertile couple's attitude toward accepting these therapeutic alternatives in infertile couples. It is therefore recommended to take steps to incorporate sociocultural beliefs into the routine care of infertile couples with the help of social media to give them ability for making more pragmatic decision in relation to their chosen options.

## 1. Introduction

Assisted reproductive donations as the last treatment have caused several social, psychological, ethical, and legal controversies and have created the questions and problems. As a result, many infertile couples do not use them (1–5). In Iran, although the infertile couples do not have any legal limitations to use these technologies, not all infertile couples use these techniques (6). In the study of Baykal and co-worker on 368 infertile Turkish women, only 23.3% of the study subjects accepted oocyte donation, 3.4% sperm donation, and 15.1% surrogacy as a method of infertility treatment (7). Also, Mohler, Richter Purewal, and Akker have reported similar results (8–10). Given that only about half of the infertile couples around the world are seeking treatment, this question suggests what factors may affect the seeking of treatment by these couples (6). Some part of people encountering with infertility is affected by the Sociocultural beliefs of the community in which they live. From the Sociocultural beliefs affecting fertility, certain behaviors can be noted about fatalism, traditional beliefs, government and religious leaders' support, patriarchal ideas for survival and generation continuing, and low chances of remarriage of an infertile woman (11). The infertile couples seeking to have children through reproductive donation may experience apparent bias and prejudice not only from the community but also from the family, relatives, and friends (6).

According to the results of Latifnejad Roudsari, infertile couples are confronted with social problems including outcomes related to impaired gender roles, stigma, and social isolation (12). The fear of spouse remarrying among Asian and African women, especially in rural areas, has been the main motivator for treatment. Great's study in Mozambique and Greil's study in South Africa and Zimbabwe have noted women referring to traditional healers and herbal medicine vendors (11, 13).

As it is mentioned, there are different beliefs and views about the reproductive donation and some of them are severely negative so much so that they may cause to ignore these medical methods. It should be noted that the medical community cannot be separated from the society, and it is required that mentioned professional team have had a comprehensive view in understanding, assessment, and management of infertile couples (14, 15). The introduction and condition of community-based care is also an attention to the individual, psychological, social, cultural, and spiritual needs (16). This is possible in the light of a broad understanding of the disease in the context of culture and society (14).

Given the importance of this topic and that no study was found about the relationship between sociocultural beliefs and infertile couples' attitude toward reproductive donation, this study was designed for the first time in Iran to determine the relationship between sociocultural beliefs and infertile couples' attitude toward reproductive donation in 2010 in Mashhad, Iran.

## 2. Materials and Methods

This was a correlational descriptive study on infertile couples referred to Milad Infertility center in Mashhad for diagnosis, treatment, and/or counseling, who were selected using convenience sampling.

The inclusion criteria included being Iranian Muslim, having a minimum literacy requirement, not using narcotics, not having a stepchild, and not suffering from a severe medical illness. To calculate the sample size, due to the lack of similar studies in this context, a pilot study was used. Hence, the correlation between the sociocultural beliefs and attitude toward reproductive donation was measured on 10 infertile couples (10 women and 10 men) who met the inclusion criteria. Then, using the formula for correlational studies and the calculated correlation coefficient and considering 0.99 (α = 0.01) degree of confidence and power 0.99 (β = 0.01), a sample size of 115 couples (230 women and men) was calculated.

The tools used for data collection in this study included a demographic and infertility-related questionnaire, a self-structured questionnaire to measure Sociocultural beliefs, as well as a questionnaire to evaluate the attitude toward reproductive donation. The demographic and infertility-related questionnaire consisted of three sections: the demographic characteristics, data about infertility and history of its treatment, and the information about the current treatment of infertility. The self-structured questionnaire for sociocultural beliefs consisted of 33 statements with a score range from 33 to 198, which measured sociocultural beliefs in five subscales. These subscales were as follows: (1) childbearing with subitems of spouse selection and traditional methods; (2) social acceptance with subitems of isolation, stigma, and family acceptance; (3) social identity with subitems of maternal or paternal role, generation survival, and prejudice; (4) religious acceptance with subitems of the sharia, reliance, destiny, and volition; and finally (5) social support with subitems of legal support, financial support, social support, job support, and media support. The higher score indicated more positive sociocultural beliefs.

The questionnaire evaluating the attitude toward reproductive donation was a modified questionnaires of Skoog, Mahram, and Khalili (17-19) focused on the attitude toward reproductive donations. The developed questionnaire included 32 statements in seven subscales with a score range from 32 to 192. The seven subscales consisted of importance of childbearing, genetic bond between parents and children, public attitude toward reproductive donation, confidentiality of the donation process, attitude toward donated oocyte, attitude toward donated embryo, and attitude toward surrogacy. The scoring of these two questionnaires were based on six points Likert scale including a range from very disagree (score 1) to very agree (score 6). A higher score indicated more positive attitude. The validity of both questionnaires on the sociocultural beliefs and attitude were determined by content validity approach. Their reliability was confirmed based on internal consistency with Cronbach's alpha coefficient of 0.887 for sociocultural beliefs and 0.757 for attitude questionnaire. A written informed consent was signed by all the subjects, and then a general description on how to respond to the questions was provided to them by the researcher. The subjects completed the questionnaire on a self-report basis and the researcher was available in case they had any questions. At the end, the researcher picked up the questionnaires and checked them for completeness.

### Ethical consideration

When the research proposal was approved by the Ethics Committee of Mashhad University of Medical Sciences with number of 88786. A written informed consent was signed by all subjects in Milad Infertility Research Center. Participants were reassured for anonymity and confidentiality of the data as well having the right to withdraw from the study without prejudice in any way.

### Statistical analysis

To describe the data, descriptive statistics including central indices and frequency distribution were used. After the data collection and data entry into computer, statistical analysis was performed using SPSS software 18 version to evaluate the relationship between sociocultural beliefs and attitude toward reproductive donation. In case of normal data distribution, Pearson correlation test and for abnormal data distribution Spearman correlation test were used. For the comparison of age, religion, place of residence, duration of marriage, duration of infertility, and duration of infertility treatment among subjects, Mann-Whitney, *T*-test were used. The confidence coefficient of 95% and a significance level of 0.05 were considered for analysis. To determine the normal distribution of continuous variables, Kolmogrov-Smirnov test was used.

## 3. Results

Participants' demographic characteristics are shown in Table I. According to the results, generally 217 patients (94.3%) were Shia and only 13 (5.7%) were Sunni. The mean duration of the marriage, infertility, and infertility treatment of samples were 7.1 ± 4.3, 5.1 ± 4.0, and 3.8 ± 4.0 yr, respectively.

Table II shows the sociocultural beliefs of infertile couples about reproductive donation. To compare the subscales of Sociocultural beliefs, mean scores were conversed to percentage with minimum 0 and maximum 100. There was no significant difference between women and men in any items of sociocultural beliefs. The highest mean score obtained from childbearing methods by the women was related to the sub-item of mate selecting. It is notable that obtaining a high score from childbearing methods means that participants had the stronger belief to reproductive donation than spouse selection and traditional methods. In men, the highest mean score obtained from all the sub-scales of social support.

**Table 1:** Sociodemographic characteristics


**Individual characteristics (n = 230)**	**Women (n = 115)**	**Men (n = 115)**
Mean age	28.8 + 6.0 (19-45)*	33.9 + 4.7 (22-45)*
Religion	
Sunni	6 (5.2%)	7 (6.1%)
Shia	109 (94.8%)	108 (93.9%)
Education	
Elementary school	7.8%	3.5%
Guidance School	14.8%	16.5%
High school and diploma	42.6%	47.8%
Advanced Diploma & BS	33.9%	30.4%
Masters	0.9%	1.7%
Inhabitance	
Village	13 (11.3%)	14 (12.2%)
City	102 (88.7%)	101 (87.8)
Materniity duration	6.9 + 4.3 (1-24)*	7.2 + 4.4 (1-22)*
Infertility duration	5.2 + 4.0 (1-22)*	5.0 + 4.0 (1-22)*
Treatment duration	3.7 + 4.1 (1-20)*	3.9 +3.8 (1-22)*
*Mean age (range)		

**Table 2:** The mean score of sociocultural biliefs' subscales in infertile men and women


**Sociocultural Subscales**						
**Group**	Childbearing methods*	Social acceptance*	Social identity**	Religious acceptance*	Social support**	Total*
Women	86.2 ± 14.6	59.2 ± 18.9	71.5 ± 12.8	64.0 ± 20.2	86.3 ± 14.6	67.7 ± 11.5
Men	70.6 ± 13.2	60.0 ± 18.7	65.4 ± 20.7	68.4 ± 17.9	84.7 ± 14.5	67.6 ± 12.4
	p = 0.59 t = -0.540	p = 0.754 t = 0.313	p = 0.622 Z = -0.493	p = 0.124 t = -1.543	p = 0.319 Z = -0.996	p = 0.928 t = 0.090
Note: *Data presented as mean ± SD; *T*-test						
**Data presented as mean ± SD; Mann-Whitney						

The lowest obtained mean score was related to the social acceptance component in women and men. The lower score here indicated the weaker beliefs toward donated methods that is influenced by participants' beliefs about the concepts of isolation, stigma, and family acceptance. Based on the results, there was a significant difference between the studied women and men in terms of subitem of spouse selection (p = 0.045), family acceptance (p = 0.046), and sharia (p = 0.041); the women had a more positive perception of spouse selection and sharia and men had a more positive perception of family acceptance. Also, the highest mean score in the infertile women and men was related to the subcomponent of financial support (94.0 ± 14.9, 92.3 ± 14.2%, respectively) and the lowest mean score was related to the subcomponent of isolation (55.6 ± 20.5 and 57.2 ± 18.9% respectively).

According to the results of the independent *t*-test, no significant difference was found between attitude of men and women. 73% of infertile women and 72.3% of infertile men had relatively positive to completely positive attitude toward these techniques. In terms of the attitudes' components, the results of the independent *t*-test showed that a significant difference was found between men and women in terms of the importance of childbearing (p = 0.005), so that the importance of childbearing in infertile women was 6.5% more than infertile men. Also, based on the results of the Mann-Whitney test, there was a significant difference between infertile men and women's attitude toward the privacy of donation process (p = 0.044), so that 4.8% more infertile men than infertile women disagreed about the privacy of donation process. About the other components of attitude, there was no significant difference between the two groups of men and women.

Based on the results of the Pearson or Spearman's correlation coefficient there was a significant direct relationship between all Subscales of Sociocultural beliefs and attitudes of infertile men toward reproductive donation (Table III). Also significant and direct relationship was found between the variable of awareness in women (p = 0.003, r = 0.273) and men (p = 0.003, r = 0.278) with a mean total score of attitude toward reproductive donation.

**Table 3:** The correlation between attitude and sociocultural beliefs' score in infertile men and women


	**Attitude**			
	**Men**		**Women**	
**Sociocultural beliefs' subscales**	**P**	**R**	**P**	**R**
Childbearing methods	0.000	0.403	0.029	0.230
Social acceptance	0.000	0.414	0.090	0.159
Social identity	0.002	0.283	0.278	0.102
Religious acceptance	0.002	0.672	0.000	0.652
Social support	0.000	0.528	0.000	0.511
Total score	0.000	0.604	0.000	0.380

The results of the ANOVA test showed that the mean total score of men's attitude toward studied techniques was significantly different in three income groups with income less than enough, enough, and more than enough (p = 0.016). Based on the results of Tukey test showed that this difference in attitude was found between the income groups of enough and more than enough (p = 0.021), so that the mean attitude score of infertile men who had enough income was 14% more than the infertile men with more than enough income. Qualitative variables along with quantitative variables that had an influence on the women's (awareness) and men's (awareness and income) attitude along with the main variable of Sociocultural beliefs were entered to the general linear regression model, and the analysis showed that none of these variables had a confounding effect on the attitudes of infertile couples. The variables of awareness (p = 0.000), educational level (p = 0.032), and place of living (p = 0.011) for women and awareness (p = 0.000) for men had a significant relationship with a mean total score of Sociocultural beliefs. Based on the results of Spearman correlation coefficient, a significant direct relationship was found between the level of education and the mean of the total score of Sociocultural beliefs (p = 0.032, r = 0.200), so with the increase in education level, the score of Sociocultural beliefs also increased.

Based on the results of the independent *t*-test, a significant difference was observed between the mean score of Sociocultural beliefs of urban and rural women (p = 0.011). It means that the mean score of Sociocultural beliefs of urban women was 14% more than the rural-resident women. Based on the results of the Pearson correlation coefficient, a significant direct relationship was found between awareness and Sociocultural beliefs of the infertile women (p = 0.000, **r = 0.379).

The results of Pearson correlation coefficient showed that there was a significant direct relationship between the mean score of awareness and the mean total score of Sociocultural beliefs in infertile men (p = 0.000, **r = 0.437). All qualitative along with quantitative variables affecting the women's (awareness, education, and place of residence) and men's (awareness) Sociocultural beliefs were entered into the general linear regression model that showed that none of these variables have a confounding effect on the Sociocultural beliefs of infertile men and women in this study.

## 4. Discussion

This study was conducted with the aim of determining the relationship between infertile couples' Sociocultural beliefs and their attitude toward reproductive donation. The results showed that there was a significant direct relationship between infertile couples' Sociocultural beliefs and their attitude toward these procedures. The attitude of men was affected by all components of Sociocultural beliefs including childbearing methods, social acceptance, social identity, religious acceptance and social support. However, the attitude of women was only affected by the components of methods of childbearing, social support, and religious acceptance. This can explain the higher correlation between Sociocultural beliefs and attitude in men rather than women.

Given that the previous studies have evaluated the attitude of infertile individuals indirectly through different cultural components and there was no similar study that directly evaluated the relationship between Sociocultural beliefs and attitude, therefore at first, we discuss the relationship between the attitude and each component of Sociocultural beliefs separately. In the present study, the “methods of childbearing” as a component of Sociocultural beliefs had a significant direct relationship with the attitude of infertile couples. With regard to the subcomponents of childbearing methods, the belief of infertile men and women about “second spouse selection” was significantly different, so that infertile men had more desire for second spouse selection than infertile women. However, both women and men earned higher score in terms of belief to second spouse selection rather than the traditional methods of childbearing, in other words, infertile couples preferred the traditional methods of childbearing to second spouse selection; when their beliefs in each of these two methods decreased, their attitude got more positive toward reproductive donation.

However, Greil (11), Ola (20), Araoye (21), and Sundby (14) in their studies stated that remarriage is the most common treatment in men. Of course, these studies were performed in poor or rural areas with low socioeconomic status, which suggests that they could not pay the costs of medical treatment for infertility. In our study, infertile couples have been studied, while these studies were conducted on the general population. One of the reasons for more tendencies to traditional methods of childbearing in this study may be related to Iran being a traditional society which provides another solution to infertile couples, that is, referring to traditional methods of childbearing. Araoye (21), Yebei (22), and Seybold also referred to the acceptance of traditional methods such as Mascot among the Senegalese (23).

In the present study, the lowest belief score of infertile couples was related to “social acceptance” as another subcomponent of Sociocultural beliefs, in the way that this belief led the infertile couples to some extent of isolation, stigma, and feeling that their family does not accept these methods. This belief, in the same way, decreased the attitude score in infertile men; however, no relationship was observed between the component of social acceptance and attitude in infertile women that indicates a significant difference between infertile men and women's belief in terms of family acceptance; the men had stronger beliefs about family acceptance and were less affected by negative attitudes of their family and their wife's family, but women had more negative beliefs than men about whether these methods would be accepted by their families. The findings of Latifnejad Roudsari *et al.* (2007) showed that infertile women suffered from stigma and social pressure and the source of pressure was the intervention of their relatives or their spouse's relatives including their constant asking about the treatment process and their non-normative/strange recommendations (24). In developing countries such as Senegal, social pressure and lack of family acceptance are factors that affect the choice of treatment (23). With regard to “religious acceptance” as another component of Sociocultural beliefs in this study, infertile couples obtained a relatively high score, as the majority of couples (90.5% men and 87.7% women) relatively to completely agreed with the phrase that donated methods can be used with trust in God without worrying about the child's future. In addition, 97.4% of infertile men and 95.6% of infertile women did not consider “destiny” as surrendering to what happened and they relatively to completely agreed with the phrase that “God works through means.” In the study of Sundby, one of the measures that help infertile couples who seek treatment was visiting the holy places and holy people, which shows the trust and relying on a great spiritual power (23).

In the study of Latifnejad, the religious women had a supernatural hope for a successful pregnancy that was raised from their belief to God's mercy and miracle (24). In the present study, the belief of infertile men about “social identity” was directly, but weakly, correlated with their attitude; however, this relationship was not observed in infertile women. Among the subcomponents of social identity, the weakest belief was related to the survival of generation, in the other words, the majority of the couples were afraid of interrupting their, as mentioned in the present study, component of “social support," as the subcomponent of Sociocultural beliefs was significantly and directly correlated with the attitude of infertile couples. Also, in terms of all the subcomponents of social support (financial, career, work, media, and legal support), the participants had strong beliefs. Among the mentioned subcomponents, the infertile couples had obtained the highest score in “financial support" in the field of infertility treatment. In the study of Chliaoutakis *et al*., increased the score of financial, career, and legal support was significantly associated with an increased intention to use donated gamete and surrogacy (25). Van Den Akker in his book entitled “Infertility Guide, Diagnosis, Treatment, and Selection" emphasized that the lack of social support for surrogacy based on the public belief can provide the context for further vulnerability (26). In this regard, the opinions of various experts in different fields that are somehow related to infertility may help to develop programs for public clarification toward various aspects of using these techniques. It seems that mass media could made the negative attitudes of the community toward infertility and ARTs less important (27). Also it seems necessary for the multidisciplinary team who provide psychosocial support for infertile women including physicians, midwives, nurses, psychologists and counselors, to recognize the appropriate coping strategies and encourage infertile couples to adopt them when they suffer from stresscaused by confrontation with negative social interactions (28). Latifnejad Roudsari *et al.* (2011, 2018) also suggested use of collaborative counselling approach through contribution of all infertility treatment team members to reduce the perceived infertility-related stress. It could be recommended to infertile couples as a stress management strategy in order to cope better with infertility (29, 30).

## 5. Conclusion

This study affirmed that individuals' attitude about reproductive donation is influenced by Sociocultural beliefs. The process of having a baby through these techniques is performed inside the society. It seems that building a cultural background, which helps to remove current doubts is necessary in order to help infertile couples make a decision without concerns about social pressures. It is therefore recommended taking steps to incorporate sociocultural beliefs into the routine care of infertile couples with the help of social media to give them ability for making more pragmatic decision in relation to their chosen options. Among these, the infertile couples had a strong belief in the role of government agencies for social support, therefore, health policy-makers should pay attention to this important issue in their strategic planning.

##  Conflict of Interest

No conflict of interest.
